# Aminodextran Coated CoFe_2_O_4_ Nanoparticles for Combined Magnetic Resonance Imaging and Hyperthermia

**DOI:** 10.3390/nano10112182

**Published:** 2020-11-02

**Authors:** Sumera Khizar, Nasir M. Ahmad, Naveed Ahmed, Sadia Manzoor, Muhammad A. Hamayun, Nauman Naseer, Michele K. L. Tenório, Noureddine Lebaz, Abdelhamid Elaissari

**Affiliations:** 1 Polymer Research Lab, School of Chemical and Materials Engineering (SCME), National University of Sciences and Technology (NUST), H-12 Sector, Islamabad 44000, Pakistan; sumera.phd@scme.nust.edu.pk; 2 Department of Pharmacy, Quaid i Azam University, Islamabad 45320, Pakistan; natanoli@qau.edu.pk; 3 Department of Physics, COMSAT University, Islamabad 44000, Pakistan; sadia_manzoor@comsats.edu.pk (S.M.); asif.hamayun@comsats.edu.pk (M.A.H.); 4 Department of Cardiology, King Edward Medical University, Lahore 5400, Pakistan; n_naseer@bahriahospitals.com; 5 Department of Chemistry, State University of Ponta Grossa, Av. Gen. Carlos Cavalcanti, 4748, CEP 84030-900 Ponta Grossa, Paraná, Brazil; mkltenorio@pq.cnpq.br; 6 Univ Lyon, University Claude Bernard Lyon-1, CNRS, LAGEPP-UMR 5007, F-69622 Lyon, France; noureddine.lebaz@univ-lyon1.fr (N.L.); abdelhamid.elaissari@univ-lyon1.fr (A.E.)

**Keywords:** magnetic particles, functionalization, adsorption, in vitro diagnosis, theranostic, MRI

## Abstract

Aminodextran (AMD) coated magnetic cobalt ferrite nanoparticles are synthesized via electrostatic adsorption of aminodextran onto magnetic nanoparticles and their potential theranostic application is evaluated. The uncoated and aminodextran-coated nanoparticles are characterized to determine their hydrodynamic size, morphology, chemical composition, zeta potential and magnetization. The aminodextran containing cobalt ferrite nanoparticles of nanometer size are positively charged in the pH range from 3 to 9 and exhibit saturation magnetization of 50 emu/g. The magnetic resonance imaging (MRI) indicates capability for diagnostics and a reduction in intensity with an increase in nanoparticle amount. The hyperthermia capability of the prepared particles shows their potential to generate suitable local heat for therapeutic purposes. There is a rise of 7 °C and 9 °C at 327 kHz and 981 kHz respectively and specific absorption rates (SAR) of aminodextran-coated nanoparticles are calculated to be 259 W/g and 518 W/g at the given frequencies larger than uncoated nanoparticles (0.02 W/g). The development of novel aminodextran coated magnetic cobalt ferrite nanoparticles has significant potential to enable and improve personalized therapy regimens, targeted cancer therapies and ultimately to overcome the prevalence of nonessential and overdosing of healthy tissues and organs.

## 1. Introduction

To timely diagnose and treat cancer is among most the prevalent issues faced by biomedical research presently. Cancer is considered a problematic disease to treat because of hurdles in prognosis and diagnosis. Moreover, conventional anticancer therapies come with several side effects. In the recent past, efforts have been dedicated to develop novel nano-magnetic materials for various potential applications [1,2]. In this context, the development of magnetic nanoparticles (MNPs) for diagnosis (non-invasive or minimally invasive) and therapy, such as carriers for magnetic resonance imaging (MRI), delivery of drug, hyperthermia, among others, has attracted great attention [3,4].

For the biomedical use of MNPs, they should present superparamagnetic behavior at room temperature. This helps them to avoid coagulation and vessel embolism while being used in an in vivo system. Furthermore, they should also present stability, biocompatibility in biological media, specific targetability and ability to encapsulate that confirm appropriate cells or tissues interaction [5]. In this context, for in vivo applications, the encapsulation of magnetic nanoparticles within a biocompatible polymer is essential [4,6], during or after the preparation process to prevent agglomeration [7].

Due to their exceptional physical characteristics, hybrid magnetic nanoparticles can be also applied as multifunctional materials, with the potential to be used in diagnosis and therapy, simultaneously [8]. This newly emerging field, called theranostics, has generated high interest, especially in cancer treatment [9,10].

Magnetic nanoparticles help protons of the surrounding medium to spin and relax at a faster rate that leads to inhomogeneity in an externally applied magnetic field [11]. Owing to this property, MNPs are utilized for diagnosis application as contrast agents for imaging, providing images with high spatial resolution [12]. MNPs show high Brownian fluctuations and rotation of magnetic moments in the crystal lattice (Néel fluctuation) in an alternating (AC) magnetic field. Both fluctuations induce generates heat known as hyperthermia [13]. Their dual role permits them to be used for targeting cancer cells for diagnosis, for therapy (release and delivery of drug, hyperthermia) and for observing the heat response.

Spinel cobalt ferrites (CoFe_2_O_4_) MNPs are being extensively investigated and have been proposed for biomedical applications [14,15]. Nanoparticles of CoFe_2_O_4_ are of great importance for their inimitable optical, magnetic, electronic and physical properties which include large magnetocrystalline anisotropy, large magnetostrictive coefficient, high Curie temperature and exceptional chemical stability [16]. For instance, cobalt ferrite nanoparticles are investigated thoroughly as suitable candidates in diagnosis and therapy (theranostics) than magnetite and maghemite nanoparticles [17,18]. They are used to enhance the response of signal in MRI, as hyperthermia agents, for bioseparation and drug delivery. Numerous approaches have been used for the synthesis of cobalt ferrite nanoparticles since their size, shape and morphology are closely associated with the preparation methods [19,20]. CoFe_2_O_4_ nanoparticles having a size between 35 to 40 nm exhibit exceptional stability in aqueous dispersion at physiological pH devoid of any variation in zeta potential and hydrodynamic size [21]. The specific control of the structure and composition of CoFe_2_O_4_ makes them useful in various biomedical and technological applications. CoFe_2_O_4_ particle size increases with a rise of annealing temperature because of the production of additional metal ions that leads to increases in the particle sizes. The particle agglomeration is due to the growing distribution of particle size at higher temperature [16]. The increase in saturation magnetization along with cobalt ferrites nanostructures size is related to their biomedical applications [22]. Due to tunable and impressive magnetic properties, CoFe_2_O_4_ could be used in MRI as an enhancer of the relaxation process and as heat producers in hyperthermia. In recent times, it has been revealed that nanoparticles of CoFe_2_O_4_ with a size greater than 5 nm possess high potential for MRI imaging displaying effectual shortening of T2 relaxation time [23]. The heating efficiency of CoFe_2_O_4_ reduces with the increasing size of nanoparticles because of the magnetic interactions of CoFe_2_O_4_ nanoparticles [16]. The smaller the particle size and the higher the field intensity, the greater was the temperature rise that resulted in better heat induction in nanoparticles. Nevertheless, it is still quite interesting and challenging to improve and enhance the magnetic properties of nanoparticles to attain greater signal sensitivity for improved contrast in imaging and for increasing the heating efficacy in hyperthermia.

Synthetic and natural polymers, organic surfactants, inorganic compounds and bioactive molecules have been extensively used as a surface coating for nanoparticle functionalization, improving its stability, pharmacokinetics, biodistribution and biocompatibility [4,6]. Aminodextran (AMD) is one of the most promising nontoxic polymers used for coating a wide variety of theranostic agents due to their excellent physico-chemical properties and physiological acceptance. AMD is gaining prominence in the biomedical field since magnetic nanoparticles coated with AMD have shown improved stability and tend to stay in circulation for relatively long periods. The AMD coating also exhibited good potential for in vivo biomedical diagnosis applications as demonstrated by their higher T2 contrast-ability compared to Gd in magnetic resonance imaging (MRI) [8]. Also, AMD provides external controllability of magnetic heat generation by magnetic nanoparticles and can be utilized not only for therapeutic hyperthermia of cancer but also for controlled release of cancer drugs through the application of an external magnetic field [3,4].

In the present investigation, an adapted sol-gel method was used for the preparation of amine-functionalized CoFe_2_O_4_ nanoparticles of potential theranostic application. Bare and functionalized nanoparticles were characterized further for their physicochemical, magnetic, structural and morphological to study the effect of AMD incorporation on the results. Aminodextran-coated magnetic cobalt ferrite nanoparticles were prepared via the layer-by-layer assembly and, its potential application in MRI and hyperthermia were also evaluated.

## 2. Materials and Methods

### 2.1. Materials

Cobalt nitrate Co (NO_3_)_2_·6H_2_O (98%) and ferric nitrate, Fe (NO_3_)_3_·9H_2_O (98%), Sodium periodate (NaIO_4_, 99.8–100.3%), 1.6-hexamethylenediamine (>98%) and Sodium borohydride (NaBH_4_, 98%) were acquired from Sigma Aldrich, Darmstadt, Germany. Dextran T40 was purchased from AppliChem PanReac, Darmstadt, Germany. All chemicals were used as received.

### 2.2. CoFe_2_O_4_ Nanoparticles Preparation

Modified sol-gel method was utilized for the preparation of nanoparticles of cobalt ferrite [24,25]. Saturated metal nitrate solution (1:2, Co:Fe ratio) was prepared and added to a dilute aqueous solution of PVA (10% *w/v*) in a metal: monomeric PVA unit ratio of 1:12. The solution obtained was kept at room temperature and maintained under stirring for 2 h. It is then heated to 250 °C, until total evaporation of water. Calcination of the precursor used for synthesis for 4 h at 400 °C is carried out to CoFe_2_O_4_ nanoparticles.

### 2.3. Synthesis of Aminodextran (AMD)

The AMD was prepared using the method described by Mouaziz et al. [26]. After dissolving Dextran T40 (10 g) in a buffer solution of sodium acetate (50 mL, pH = 6.5) at room temperature, NaIO_4_ (5.28 g) was taken to this solution for the oxidation process under dark and stirring conditions for 2.5 h. The obtained solution was dialyzed through a membrane (d = 29 mm, Molecular weight cut off = 12,000–14,000) with 6 lit of Milli-Q water, for 20 h. Then, the oxidized dextran solution was brought to a temperature of nearly 8 °C using an ice bath before the addition of 6.31 g of 1.6-hexamethylenediamine in the liquid form in a water bath at a temperature of 45 °C. It was stirred for 1.5 h to obtain a consistent solution at this temperature. To carry out the reduction of amine functional groups, finally, 40 mL solution of sodium hydroxide (NaOH 10^−3^ M) and 4.10 g sodium borohydride (NaBH_4_) were added to the previously prepared homogeneous solution under constant stirring for 3 h at room temperature. The final solution was dialyzed for 24 h with 9 L of Milli-Q water and lyophilized (Cryonext freeze drier, model Lyo Pilote 38L) to get aminodextran as a white powder.

### 2.4. Adsorption of Aminodextran (AMD) to Magnetic Nanoparticles

22.67 mg of cobalt ferrite nanoparticles (powder) were dissolved in 3 mL Milli-Q water, under sonication 70% for 4 min. Aminodextran solutions of 10 mL with varying concentrations of 6.32; 76.50; and 125.26 mg/L were added with 3 mL/h into the prepared magnetic dispersion under mechanical stirring given in Table 1. After the addition of magnetic particles, the suspension was stirred for 15 min. This was followed by a multistep procedure that involved a separation of the AMD-coated cobalt ferrite nanoparticles using a magnetic bar, removal of excel polymer through washing and redispersion again in Milli Q water (15 mL). Prepared samples were designated with codes. For instance, the prepared sample with an initial ratio of 20 mg AMD/g is designated as sample 2.

### 2.5. Characterization Techniques

#### 2.5.1. X-ray Diffraction (XRD) of Co_2_Fe_2_O_4_

XRD analysis was done to identify the crystallinity and average crystallite size of the nanoparticles using an XRD diffractometer (STOE, Darmstadt, Germany). A small amount of powdered nanoparticles was placed in Cu KR radiation (Cu-Kα, λ = 1.54056 Å), at 30 mA and 40 kV and scanned steadily between 0° to 80° at 2θ. Several intensity data sets were acquired by recording at every spot. All the XRD peaks were analyzed and indexed using the ICDD database, relating to cobalt ferrite standards. An XRD spectrum was plotted with the help of Origin software and the average size of nanoparticles (D) was calculated using the Scherrer equation described below:

(1) $$D=\frac{K\lambda }{\beta cos\theta \;}$$

where *K* is the nanoparticles shape factor, *λ* is the wavelength of the X-rays, *β* is the full width at half maximum of X-ray diffraction peak and *θ* is the Bragg angle.

#### 2.5.2. Conductometric Titration of Aminodextran (AMD)

Conductometric titration was accomplished using an automated pH meter (Jenway IC4330, Staffordshire, UK) at room temperature. A dilute solution of particles (25 mL) with a volume fraction of 0.2% was used in a stirring vessel. NaOH solution (0.01 M) was used due to the nature of the surface groups. 0.25 mL of 1 M HCl was added to the vessel before starting the titration to get an increase in the initial slope of the curves. The amount of anime groups was calculated using the following Equation (2) [26].

(2) $$\left[{\mathrm{NH}}_{3}^{+}\right]=\frac{t_{m}.v_{t}}{m}$$

where *t_m_* is the titrant molarity (moles/L), *m* is the sample mass (g) and *v_t_* is the titrant volume (mL).

#### 2.5.3. Elemental Analysis of Aminodextran (AMD)

The elemental analysis was used to determine the chemical composition of AMD using (CNRS, Solaize). The quantity of carbon, hydrogen, oxygen and nitrogen are measured in prepared aminodextran.

#### 2.5.4. FTIR Spectroscopy

Surface properties of prepared uncoated and aminodextran-coated nanoparticles were investigated by Attenuated Total Reflection–Fourier Transformed Infrared Spectrophotometer (ATR–FTIR) (Shimadzu, Japan). All samples were desiccated afore analyses. FTIR spectrum in the range of 4000–400 cm^−1^ and 50 scans were obtained for each sample with a 4 cm^−1^ resolution.

#### 2.5.5. Zeta Potential Measurements

The electrophoretic mobility was determined by a Malvern Zetasizer (Nano ZS, Malvern Instruments, Worcestershire, UK). Smoluchowski’s equation was then employed to determine zeta potential. Nanoparticles with highly dilute concentrations were taken in 10 mM NaCl solution and averages of three measurements were taken at different values of pH varied by using HCl or NaOH.

#### 2.5.6. Measurements of Particle Size

To estimate the average hydrodynamic size (*D_h_*) of the nanoparticles in a 10 mM solution of sodium chloride, a Malvern Zetasizer (Nano ZS, Malvern Instruments, Worcestershire, UK) was used. The reported *D_h_* is the mean of three measurements at least.

#### 2.5.7. Particle Size and Morphology Analysis

Transmission Electron Microscopy (TEM, Thermo Fisher Scientific, Eindhoven, Netherland) by Philips CM120 at the “Centre Technologique des Microstructures” (CTμ) was used under 120 kV acceleration voltage. Experimentally, a droplet of the diluted aqueous solution of all samples was dispensed and dried on a copper grid covered with carbon under ambient conditions. Particle size and morphology were also studied by scanning electron microscopy (SEM, JEOL JSM 6490LA Akishima, Tokyo, Japan) (The technique has been provided in Supplementary Materials).

#### 2.5.8. Magnetization Measurements

Vibrating samples magnetometer (Automatic Bench of Magnetic Measurements, CNRS-IRC, Villeurbanne, Sherwood Scientific, Cambridge, UK) was used to study the magnetic characteristics and saturation magnetization of the nanoparticles in dry form. All the measurements were taken at room temperature.

#### 2.5.9. Magnetic Resonance Imaging (MRI) Analysis

Diagnostic potential of nanoparticles of cobalt ferrites was investigated through in vitro MRI scans utilizing a clinical 1.5 T MRI machine from Toshiba Vantage Titan, Japan. A particular amount (g) of two samples labeled as samples 1 and 3 were taken in water (10 mL) to acquire T2 weighted images. Typically, particular concentrations labeled as C1 (0.1073 g/10 mL), C2 (0.2201 g/10 mL), C3 (0.2042 g/10 mL), C4 (0.1254 g/10 mL), C5 (0.2214 g/10 mL) and C6 (0.2053 g/10 mL) were prepared in deionized water. MRI measurement features a 1 cm aperture with the FOV of 55 × 55 × 50 cm and acoustic noise was reduced using Pianissimo technology. T2 sequences (ms) were run to acquire intensity of signal using MRI field echo (FE) sequences at constant TR (repetition time) = 5000 ms and variable TE (echo Time) values (ms). The MRI scans were further analyzed by manually calculating corresponding intensities using Kpacs software (Version 1.5).

#### 2.5.10. Hyperthermia Study

For hyperthermia efficiency of the synthesized nanoparticles or magnetothermia characteristics, Nanotherics Magnetherm 1.5 induction unit was used, functioning at two frequencies and fields, f = 327 kHz, H = 17 mT and f = 519 kHz, H = 23 mT were used. The coil temperature was maintained at 12 °C by circulating the chilled water. For measurement, the ML T640 sample was taken in an Eppendorf tube, which was placed into an insulated container to assure good adiabatic conditions. At every 1 s intervals, the temperature was recorded using a Neoptix that is an optical fiber used as a temperature sensor having a resolution of 0.1 °C. The heating curves have been used to obtain the specific absorption rate (SAR) of the CoFe_2_O_4_ nanoparticles. This is the amount of heat generated per unit mass of the nanoparticles and calculated by using Equation (3) [27].

(3) $$SAR=\frac{cM}{m}.\frac{dT}{dt}$$

where *c* is the sample-specific heat, *M* is the sample total mass and *m* is the mass of magnetic nanoparticles (grams). The sample temperature profile is given by *dT/dt* as the linear slope of initial experimental data obtained by a linear fitting.

## 3. Results and Discussion

### 3.1. XRD Study of CoFe_2_O_4_ Nanoparticles

Nanocrystalline ferrites of cobalt showed diffraction maxima compatible with an inverse-spinel type structure, as observed from its X-ray diffraction profile shown in Figure S1 (Reference code: 00-022-1086). The average size of the crystalline domain determined using the (311) plane was calculated by the Scherrer Equation (1) and found to be 25 nm. All the detected reflections found planes of (220), (311), (400), (422), (511), (440) and (622) in the diffractogram of cobalt ferrite sample could be assigned to the cubic inverse spinel lattice indicating their single-phase nature.

### 3.2. Conductometric Analysis of Aminodextran (AMD)

The conductometric titration was used to determine the amount of amine groups in the aminodextran polymer (Figure S2). The concentration of amine groups [NH_3_^+^] was calculated from the intersection of three branches of the titration curve using Equation (1) that was found to be equal to 5 meq/g, equivalent to 0.705 moles of amines per mole of dextran. Consequently, this relates to one full oxidation for 5 units of dextran. The difference in two intersection points relates to the volume of base requisite to neutralize the acid groups.

### 3.3. Elemental Analysis of Aminodextran (AMD)

Elemental analysis data was used to determine the amine content in AMD polymer. Elemental analysis of AMD was C, 48.5%; H, 8.1%; O, 38.6%; N, 5.6% which is equivalent to 0.71 moles of amine per mole of dextran. Elemental analysis was in agreement with conductometric titration results. Consequently, the substitution degree of diamine in dextran was around 1/6 in comparison to a theoretically calculated value of 1/2.5 based on 100% periodate cleavage and diamine substitution.

### 3.4. FTIR Analysis

**Figure 1:**
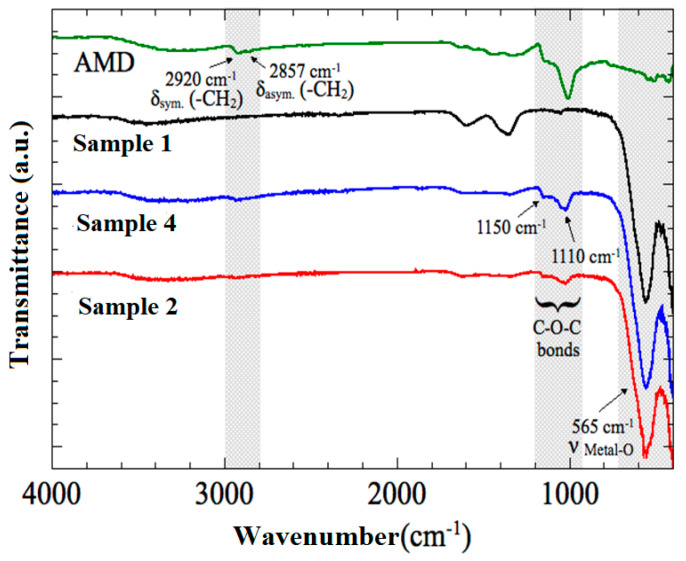
Fourier transform infrared (FTIR) spectra of sample 1 (cobalt ferrites), aminodextran (AMD) and, AMD-coated CoFe samples (sample 2 and sample 4).

Fourier transform infrared (FTIR) spectroscopy was a suitable technique for the identification of chemical functional groups present on the synthesized nanoparticles. Both uncoated and coated nanoparticles were analyzed by FTIR spectroscopy (Figure 1), to confirm the presence of amine groups (-NH_2_) onto magnetic particles surface over a range of 400–4000/cm^−1^. FTIR spectra for uncoated cobalt ferrites (sample 1) illustrates characteristic peaks at 430 cm^−1^ and 570 cm^−1^, which were due to the intrinsic vibrations of the octahedral and tetrahedral coordination compounds and lattice vibrations [28,29]. These two bands are found in almost all spinel ferrite structures. Spectra of the AMD-coated samples obtained from FTIR show the high-intensity characteristic bands of cobalt ferrites, at around 565–400 cm^−1^, related to the vibration of the oxygen-metal cation complexes present in the octahedral sites (Mocta-O) and tetrahedral (Mtetra-O), respectively [30]. Also, coated samples showed bands at around 1150 and 1110 cm^−1^, due to C-O-C bonds in the aminodextran pyranose ring and at 2920 and 2857 cm^−1^, attributed to asymmetric and symmetric stretching modes of CH_2_. Absorption bands related to the polymer in both sample 2 and sample 4 spectra confirm the presence of amine groups (-NH_2_) due to successful adsorption of aminodextran onto the surface of sample 1.

### 3.5. Size and Surface Morphology

**Figure 2:**
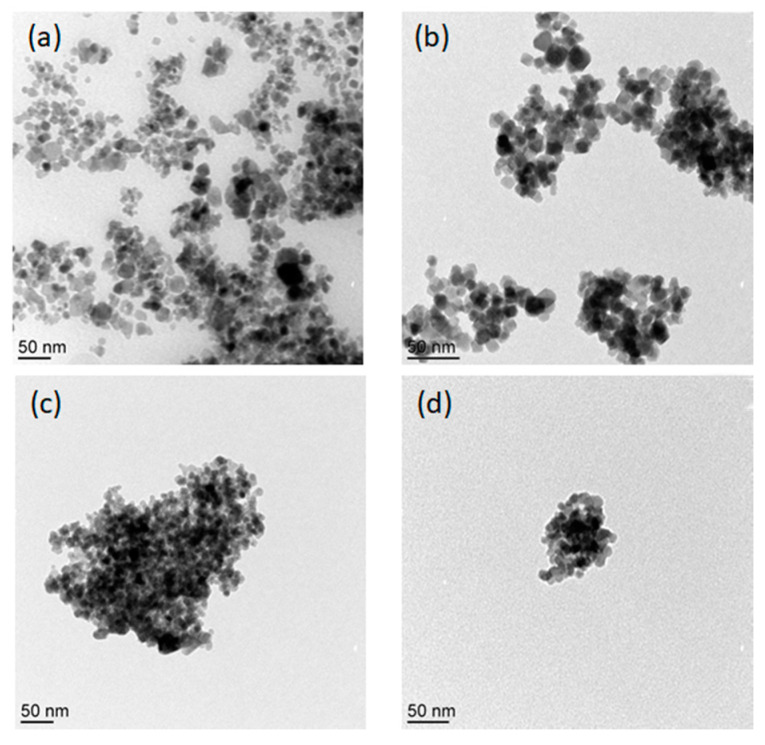
Transmission electron microscopy (TEM) images of coated and uncoated samples. (**a**) Sample 1, (**b**) Sample 2, (**c**) Sample 3, and (**d**) Sample 4.

To examine the size and morphology of uncoated and coated nanoparticles, scanning electron microscope and transmission electron microscopy coupled were performed. Figure 2 shows transmission electron microscopy (TEM) images of 1, 2, 3 and 4 samples and revealed nanoparticles with an average size between 10 and 50 nm. Likewise, scanning electron microscopic (SEM) images (Figure S3) of 1 and 4 samples were also obtained and showed nanoparticles of uniform size and shape. No difference in terms of size and shape was found between both samples as it can be seen in SEM and TEM images. Spherical/cubic particles were observed with some extent of aggregation without any evidence of the polymer shell on aminodextran(AMD)-coated nanoparticles, due to the low density of interfacial shell of AMD, as stated earlier in the literature [31]. The images revealed that there is no effect of polymer coating on the nanoparticle crystallite size which leads to the agglomeration of the coated sample particles. The aggregation due to the magnetic and dipolar interactions between the particles which results in wide hydrodynamic size distribution (Figure S4) and agreement with previously reported data [32]. SEM and TEM images are also consistent in terms of aggregation of the samples, independent of AMD-coating. Both magnetic particles (coated and uncoated) showed wide particle size distribution due to the formation of some aggregates, as observed by TEM images.

Hydrodynamic diameters (Figure S4) of uncoated nanoparticles showed two average sizes observed in sample 1 correspond to two peaks in its hydrodynamic particle size distribution. After surface modification, the average particle size showed a decrease in the hydrodynamic diameter from an average size of 377 nm for cobalt ferrite nanoparticles to around 200 nm for AMD-coated nanoparticles. Wide size distribution was apparent in all the samples. The AMD adsorption induced good colloidal stability of the prepared dispersions compared to free AMD particles.

### 3.6. Electrokinetic Study

**Figure 3:**
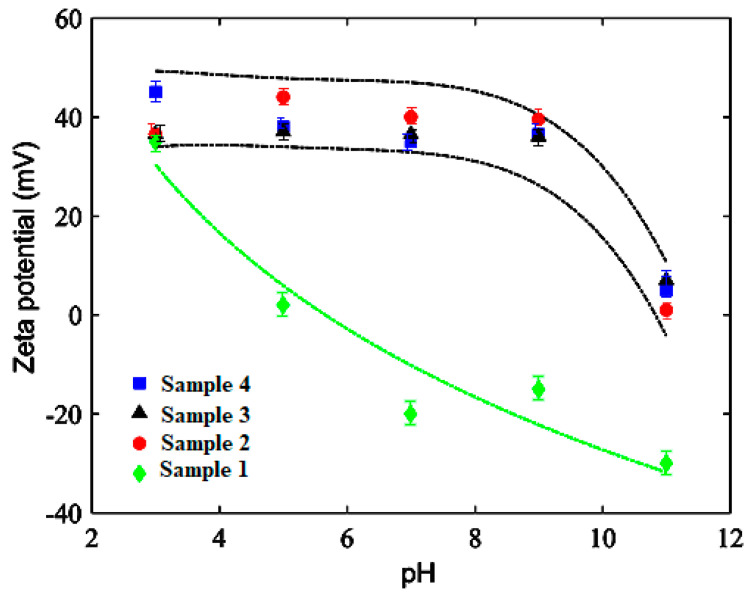
Zeta potential of uncoated cobalt ferrite nanoparticles (sample 1) and aminodextran-coated cobalt ferrite nanoparticles (samples 2, 3 and 4) as a pH function.

This particle charge variation as a function of pH determines the colloidal stability which was evaluated by zeta potential measurement. Coated cobalt ferrite nanoparticles synthesis was based on water-soluble aminodextran (polymer) adsorption at solid-liquid interfaces of uncoated nanoparticles dispersed in water. The adsorption of AMD results from the establishment of polyelectrolyte interaction between aminodextran (polycation) and cobalt ferrite particles (negatively charged). The modification of surface from negatively charged cobalt ferrites into positive AMD-coated magnetic nanoparticles was examined by measurement of zeta potential at various pH of 10 mM NaCl solution for magnetic nanoparticles and various aminodextran concentrations, as shown in Figure 3.

The uncoated nanoparticles are positively charged below pH 5.2 and negatively charged above due to possible hydroxyl group’s protonation present on the surface as reported for inorganic oxide nanoparticles. The uncoated nanoparticles showed superior stability due to its higher electrophoretic mobility at low and high pH values [33]. The coated nanoparticles are positive in the pH 3 to 9 range due to the protonation of amine groups, causing positive quaternary ammonium [-NH_3_^+^] cations formation. The pKa s is around pH 10 of the primary amine group. In this context, at pH>10, amine groups are not positively charged [-NH_2_] leading to a decrease particle surface charge.

pH dependence of surface charge of uncoated and coated particles was recognized by electrophoretic mobility measurements. It gives information regarding the predicted stability of the system in water. As clearly evidenced, uncoated nanoparticles are charged positively below pH 5.2 and negative above. With AMD-coating, latex magnetic particles showed to be positively charged at pH from 3 to 9. At a pH of 11, samples zeta potential showed to be dependent on the AMD amount, tending to the zeta potential value of the uncoated nanoparticles, when reducing the AMD content. It was concluded from the results that the colloidal stability of all the samples decreases with an increase in pH value.

### 3.7. Magnetic Properties

**Figure 4:**
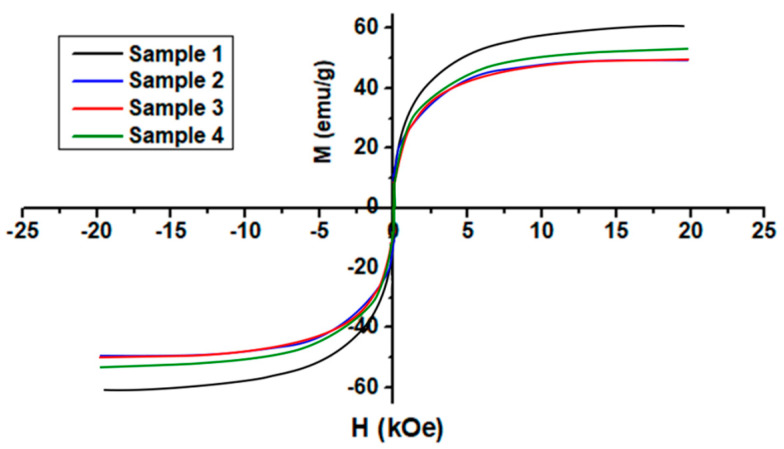
Magnetization curves of CoFe_2_O_4_ nanoparticles (Sample 1) and aminodextran coated nanoparticles (Sample 2, 3 and 4) at room temperature.

The magnetic behavior of coated and uncoated cobalt ferrite nanoparticles was displayed in Figure 4. Magnetic nanoparticles retain superparamagnetic behavior even after AMD modification. The saturation magnetization (Ms) was perceived to be close to 60 emu/g at an applied field of 20 kOe at 300 K for sample 1, as also reported in the literature [16,34]. The investigations showed that all samples of nanoparticles exhibit magnetic behavior. This value was reduced to around 50 emu/g with surface conjugation of the magnetic nanoparticles with AMD.

In samples of coated nanoparticles, the magnetization value decreases due to organic ligands present on the particle surface. Aminodextran presence on the particles consequently reduced the amount of magnetic material over the total amount. The lessening in the saturation magnetization of the coated nanoparticles can be attributed to the coating cooperativity that affects the interaction of spins located in the regions on the nanoparticles surface [35]. Cobalt ferrite nanoparticles are coated with AMD to make them water-dispersible also result in a reduction of saturation magnetization due to the dilution effect from adsorbed water.

The saturation magnetization of sample 1 was found to lower than the reported values for the bulk samples (80 emu/g) due to surface defects, morphology or disorder canting spins [34]. The surface defects are the results of finite-size scaling of nanocrystallites, which in turn leads to a non-collinearity of magnetic moments on their surface. After AMD capping, Ms values were reduced to 50 emu/g due to the presence of a nonmagnetic inactive layer on the surface of nanoparticles. AMD interfacial layer is considered a dead layer that causes the quenching of surface moments and ultimately reduction of magnetization. Saturation magnetization increases with particle size but in this work, the wide size distribution in all samples also leads to a reduction of Ms values.

### 3.8. MRI Diagnostics via Contrast Enhancement

**Figure 5:**
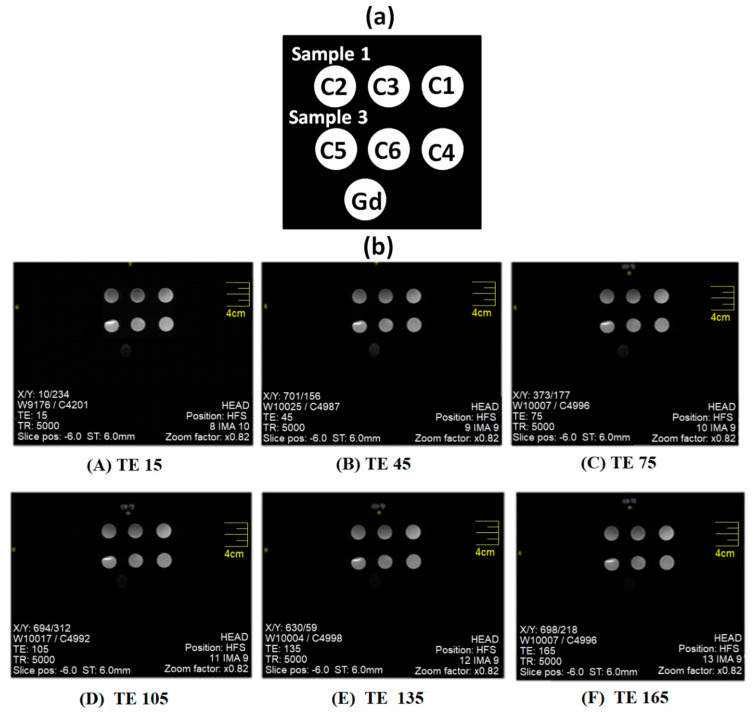
(**a**) Samples placement inside an MRI machine of the magnetic nanoparticles of samples 1 and 3 along with Gd as reference vials. See Table 2 for various samples concentrations samples 1 and 3 and Gd and (**b**) Acquisition of T2 images for magnetic nanoparticles of samples 1 and 3 along with Gd as reference vials at TR 5000 and various TE values.

**Figure 6:**
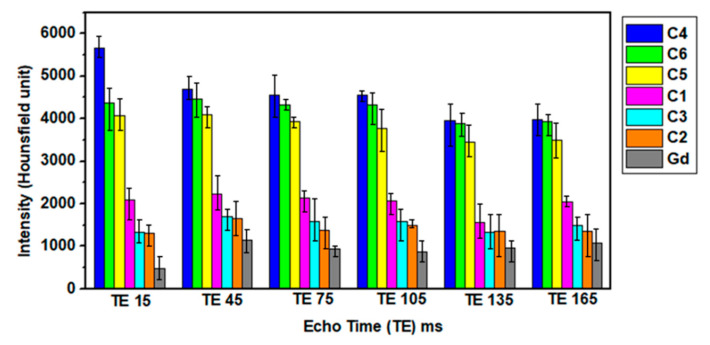
T2 intensities of magnetic nanoparticles samples for two concentrations of sample 1, sample 3 and Gd.

T2 contrast agents based on colloids containing magnetic nanoparticles can induce decay in signal intensity and shortening of spin-spin relaxation time to exhibit excellent MRI diagnostics characteristics [36,37,38]. Variation in T2 intensities for specific tissues enables differentiation from each other since water can appear lighter as compared to fat tissues [39]. Furthermore, magnetic contrast agents produce a noticeable change in the relaxation time of hydrogen atoms present in body tissues. T2 measurements were acquired through the acquisition of MRI scan images. Various samples prepared with different concentrations of magnetic nanoparticles (C1, C2, C3, C4, C5 and C6) were employed for MRI measurements summarized in Table 2. The experimental sample layout employed for measurement is presented in Figure 5. At various TE values, T2 weighted images obtained for the magnetic nanoparticles and commercially available Gd samples are presented in Figure 6. ROI (region of interest) intensity values were manually drawn in MRI images obtained at constant TR values (5000 ms) but variable TE values to get the optimal T2 signal intensity at a particular TE.

Figure 5 and Figure 6 indicate that both samples generate higher negative signal intensity to assist in visualizing cells that appear bright. This observation supports their better contrast capability as sensitive MRI contrast agents (CAs) [40]. MNPs in colloids are capable to undergo higher local inhomogeneity as compared to that of the Gd magnetic field, externally applied. In addition to hydrophilicity, surface functionality of aminodextran-coating in the magnetic colloids is also vital in the reduction of spin-lattice relaxation time. The hydrophilicity of colloids allows proximity of more water molecules to nanoparticles that ultimately affect the relaxivity. The nature of coatings (hydrophobic or hydrophilic) on nanoparticles strongly effect hydration degree to enhance their MRI imaging ability [41].

It was perceived that with an increase in the concentration of iron from lower to higher values, signal intensity decreases [23]. Relaxation rates of protons intensely depend on the coating hydrophilicity and the agglomeration of nanoparticles due to inter particle’s magnetic interactions. The shortening of T2 relaxation is caused by the functionalization of nanoparticles by aminodextran coating that lessens particle agglomeration by increasing inter-particle spacing as apparent from Figure 5 and Figure 6. The signal intensity of T2 images decreases with an increase in the iron concentrations and these results are in agreement with previously reported literature of MRI data [32]. A higher negative contrast was generated by specimens with higher iron concentrations. The combination of surface functionalities along with iron oxide enhances the hydrophilicity to allow closeness of molecules of water to generate short T2 relaxation time as mentioned earlier. The modification of nanoparticles with aminodextran-coating produced a greater hydration effect and therefore greater closeness of water molecules to the magnetic colloids that lead to reducing the spin-lattice relaxation time. Sample 1 containing only bare cobalt ferrites without AMD coating, showed greater negative signal intensity as compared to coated sample 3. In both samples, T2 values were significantly shortened and had a strong dependence on iron concentration [23]. The darkness in MRI images intensifies as the concentration goes higher for both samples (1&3). Compared to these samples (1&3), T2 weighted images of Gd appeared to be darker. This can be due to the agglomeration of cobalt ferrites nanoparticles in samples 1 & due to the non-uniform distribution of nanoparticles in the dextran matrix that strongly effects the relaxation rates [26].

### 3.9. Hyperthermia Study

**Figure 7:**
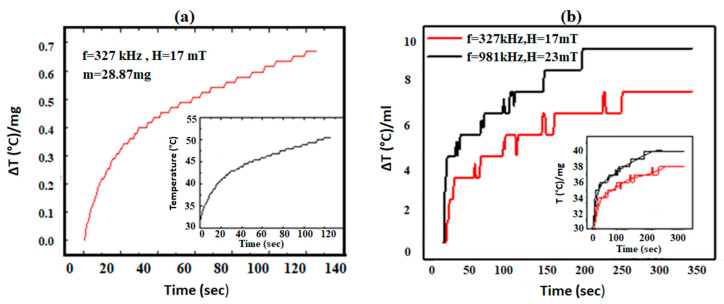
(**a**) Magnetic hyperthermia measurements were taken on CoFe_2_O_4_ nanoparticles (sample 1) displayed as a temperature change ΔT of a sample per milligram and (**b**) Magnetothermia measurements on sample 3 represented as a change in temperature ΔT per ml of the dispersion. The inset shows the original heating curves. Solid lines are guided to the eye.

The heating induction unit was used to examine the heating characterization of magnetic dispersions [16]. Magnetic hyperthermia was measured on the pure CoFe_2_O_4_ (sample 1) as well as on the dispersion of sample 3 and results are displayed in Figure 7a,b. Figure 7 presents temperature change ΔT per milligram (and per milliliter) of the sample. Equation (3) was used to estimate the SAR values of sample 1 and found to be 0.02 W/g that is in agreement with the reported literature [42,43]. Figure 7b shows the magnetic hyperthermia measured on the dispersion of sample 3. The results have been normalized as a change in temperature ΔT per ml of the dispersion. The inset shows the original heating measurements. There is a perceptible decrease in the heating capability of coated nanoparticles in an applied alternating (AC) magnetic field.

Hyperthermia measurements were carried out at two field and frequency values and indicate a different degree of heating. The sample responds primarily to the change in frequency of the applied AC frequency and generates higher heating at f = 981 kHz even though the magnetic field is somewhat lower than that at f = 327 kHz. The small crystallite sizes of the nanoparticles allow them to have a short relaxation time that allows the nanoparticles to respond to higher RF frequencies.

The results show that in the case of sample 1, there is a continuous and rapid increase in temperature. There is a rise of 50 °C in 120 s. Sample 3 attains therapeutic temperatures of 42 to 47 °C from the normal body temperature of 37 °C, at both sets of RF frequencies and magnetic field amplitudes. Saturation was attained in sample 3 after a temperature rise of 50 °C in 250 s. Further quantifiable understanding of the heating efficiencies of the diverse nanoparticles can be attained by calculating SAR values [27]. The initial slope has been extracted from the heating curves to calculate the SAR of the sample in the two different field conditions. The values obtained are 259 W/g for f = 327 kHz, H = 17 mT and 518 W/g for f = 981 kHz, H = 23 mT. These values are comparable with those reported for iron oxide nanoparticles [44,45]. The high specific absorption rate of the magnetic nanoparticles showed that the prepared nanoparticles have the potential to be used for practical therapy in targeted hyperthermia.

## 4. Conclusions

These hybrid magnetic nanoparticles with different aminodextran/cobalt ferrite nanoparticles ratio (20 mg/g, 640 mg/g and 1280 mg/g) were prepared via electrostatic adsorption. FTIR spectra showed bands characteristic of aminodextran (AMD) groups that confirmed the AMD-coating on cobalt ferrite nanoparticles. AMD-coated nanoparticles showed a mean hydrodynamic size of approximately 200 nm having wide size distribution. After AMD-coating, magnetic particles showed no changes in their morphology. The stability of coated and uncoated nanoparticles decreases with an increase in pH. Uncoated nanoparticles have a positive charge below 5.2 and the coated nanoparticles are positively charged in the pH range from 3 to 9. The reduced value of saturation magnetization of coated nanoparticles (50 emu/g) as compared to uncoated nanoparticles (60 emu/g) is due to their surface modification with aminodextran. However, all the samples showed to be superparamagnetic, thus substantiated to be prospective candidates for cancer diagnosis and treatment as theranostic agents. Aminodextran coating enhanced the MRI diagnostic capability of cobalt ferrites due to an increase in hydrophilicity characteristics. A negative contrast was observed to be higher, with an increase in iron concentration. All prepared samples showed a significant heating effect at both frequencies with SAR of 259 W/g and 518 W/g indicating their suitability meant for hyperthermia applications.

## Figures and Tables

**Table 1:** Codes of different samples prepared with different ratios of aminodextran (AMD).

Sr. No.	Sample Code	Ratio of AMD/Cobalt Ferrite Nanoparticles (mg/g)
1	Sample 1	-
2	Sample 2	20 mg/g
3	Sample 3	640 mg/g
4	Sample 4	1280 mg/g

**Table 2:** Details and concentrations of samples prepared for the transmission electron microscopy (TEM) scans.

Sr. No.	Sample Code	Description	Amount in H_2_O (g/10 mL)
1	Sample 1	Solid contents = 100% powder Size: 303 nm Zeta Potential: −13.4 mV	0.1073 (C1) 0.2201 (C2) 0.2042 (C3)
2	Sample 3	Solid contents: 0.40% (*W/V*) Size: 270 nmZeta Potential: +39.4 mV Aminodextran (AMD) coated positively charged colloids	0.1254 (C4) 0.2214 (C5) 0.2053 (C6)
3	Gd	Magnevist^®^ Bayer	0.1331

## Supplementary Materials

The following are available online at https://www.mdpi.com/2079-4991/10/11/2182/s1, Section 2.5.7. Particle size and morphology analysis. Figure S1: XRD spectra of uncoated cobalt ferrite nanoparticles (Sample 1), Figure S2: Conductometric titration curve of the amino groups of aminodextran, Figure S3: SEM images of coated and uncoated nanoparticles. (a) Sample 1 (b) Sample 4, Figure S4: (a) Hydrodynamic size distribution profiles of the synthesized coated and uncoated nanoparticles and (b) Average hydrodynamic size of nanoparticles (Sample 1, 2, 3, and 4) based on intensity weighted data.
