# Supplementary material for: Aminodextran Coated CoFe2O4 Nanoparticles for Combined Magnetic Resonance Imaging and Hyperthermia
Source: Nanomaterials (Basel). 2020 Nov 2;10(11):2182. doi: 10.3390/nano10112182 (PMC7692372; doi:10.3390/nano10112182)
Supplement: Supplementary file 1 [file nanomaterials-10-02182-s001.pdf]

nanomaterials

# Supplementary Materials

# Aminodextran Coated $CoFe_2O_4$ Nanoparticles for Combined Magnetic Resonance Imaging and Hyperthermia

**Sumera Khizar [1], Nasir M. Ahmad [1,*], Naveed Ahmed [2], Sadia Manzoor [3], Muhammad A. Hamayun [3], Nauman Naseer [4], Michele K. L. Tenório [5,6], Noureddine Lebaz [6] and Abdelhamid Elaissari [6]**

1 Polymer Research Lab, School of Chemical and Materials Engineering (SCME), National University of Sciences and Technology (NUST), H-12 Sector, Islamabad-44000, Pakistan; sumera.phd@scme.nust.edu.pk (S.K.)
2 Department of Pharmacy, Quaid i Azam University, Islamabad 45320, Pakistan; natanoli@qau.edu.pk (N.A.)
3 Department of Physics, COMSAT University Islamabad, 44000, Pakistan; sadia_manzoor@comsats.edu.pk (S.M.); m.asif.hamayun@gmail.com (M.A.H.)
4 Department of Cardiology, King Edward Medical University, Lahore, Pakistan; Nauman.naseer@gmail.com (N.N.)
5 State University of Ponta Grossa, Department of Chemistry, Av. Gen. Carlos Cavalcanti, 4748, CEP 84030-900 Ponta Grossa, Paraná, Brazil; mk.lima@yahoo.com (M.K.L.T.)
6 Univ Lyon, University Claude Bernard Lyon-1, CNRS, LAGEPP-UMR 5007, F-69622 Lyon, France; noureddine.lebaz@univ-lyon1.fr (N.L.); abdelhamid.elaissari@univ-lyon1.fr (A.E.)
* Correspondence: nasir.ahmad@scme.nust.edu.pk (N.M.A.)

2.5.7. Particle size and morphology analysis

SEM was made with a Hitachi S800 FEG microscope at the “Centre Technologique des Microstructures” (CTμ) at the University of Lyon (Villeurbanne, France). An aqueous suspension of particles was diluted, poured, and dried on a flat steel holder, at room temperature. Finally, samples were sputtered with copper under vacuum. SEM images of the samples were obtained at 15 kV (accelerating voltage).

3.1. XRD Analysis

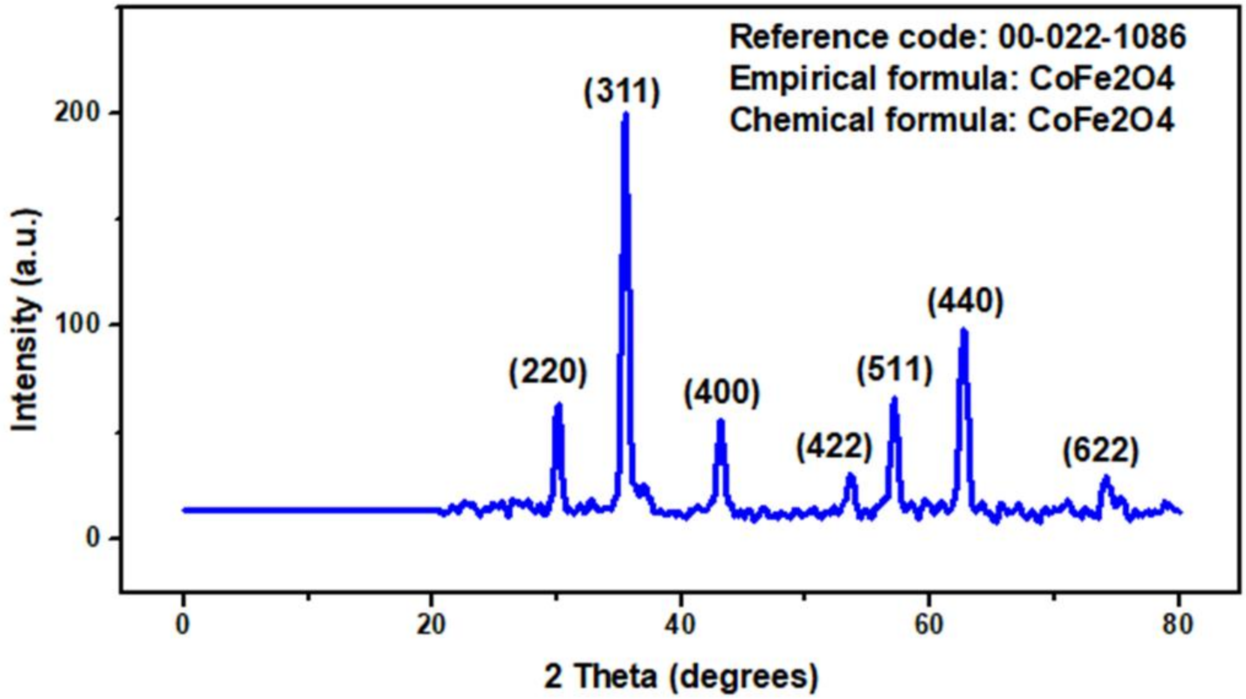


**Figure S1.** XRD spectra of uncoated cobalt ferrite nanoparticles (Sample 1).

3.2.Conductometric Analysis of AMD

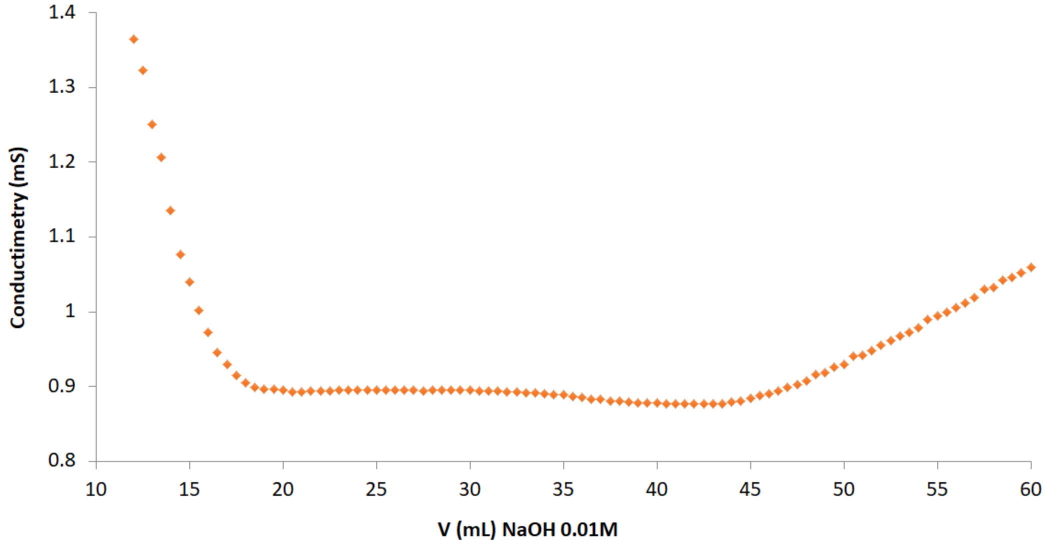


**Figure S2.** Conductometric titration curve of the amino groups of aminodextran.

nanomaterials

3.5. Size and surface morphology

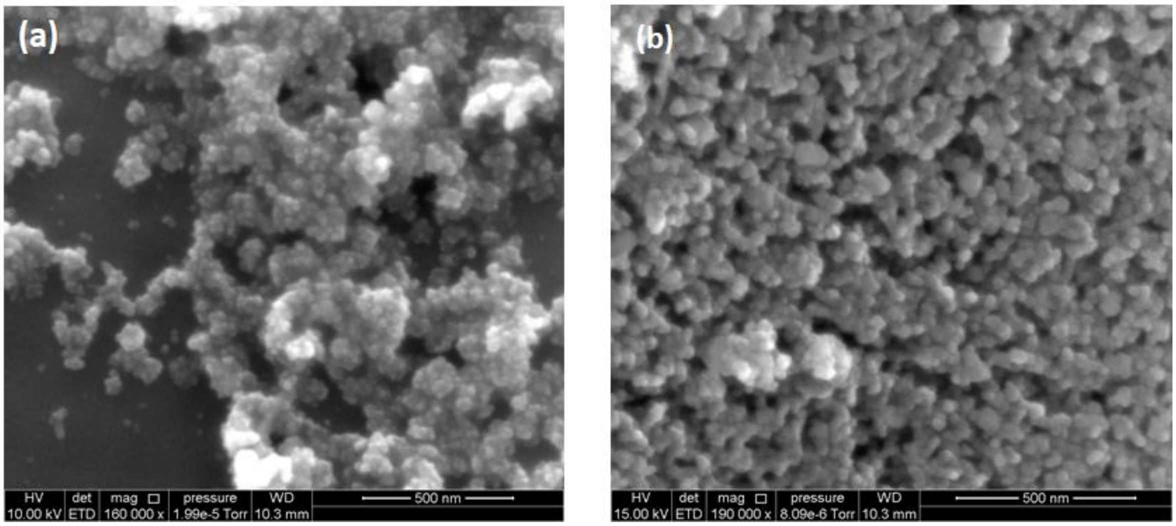


**Figure S3.** SEM images of coated and uncoated nanoparticles. (a) Sample 1 (b) Sample 4

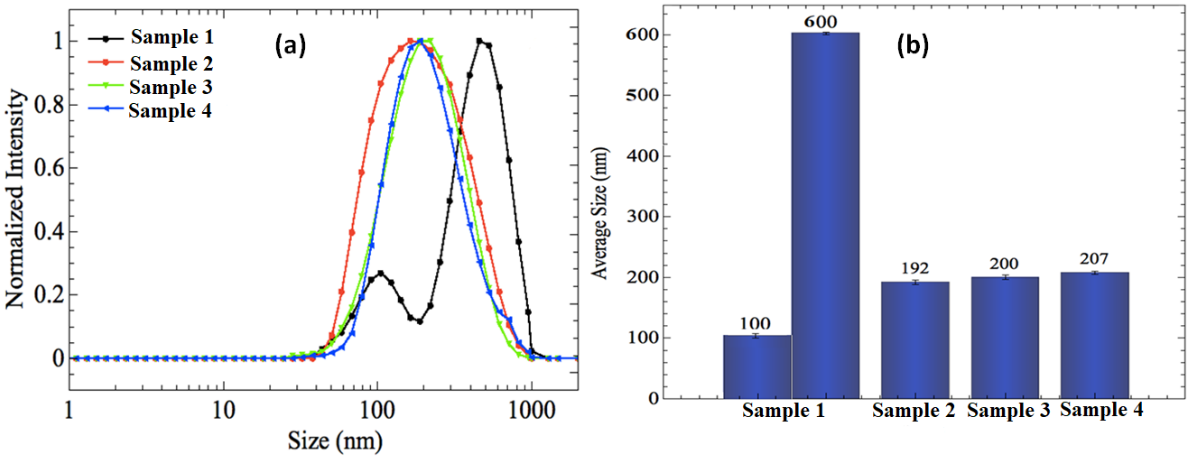


**Figure S4.** (a) Hydrodynamic size distribution profiles of the synthesized coated and uncoated nanoparticles and (b) Average hydrodynamic size of nanoparticles (Sample 1, 2, 3, and 4) based on intensity weighted data.

© 2020 by the authors. Submitted for possible open access publication under the terms and conditions of the Creative Commons Attribution (CC BY) license (http://creativecommons.org/licenses/by/4.0/).
